# Perceived and Received Dimensional Support: Main and Stress-Buffering Effects on Dimensions of Burnout

**DOI:** 10.3389/fpsyg.2019.01724

**Published:** 2019-08-02

**Authors:** Chris Hartley, Pete Coffee

**Affiliations:** Faculty of Health Sciences and Sport, University of Stirling, Stirling, United Kingdom

**Keywords:** perceived availability of support, received support, stress, sport psychology, moderation

## Abstract

Social support is an adaptive resource associated with lower levels of burnout in sport. The effects of social support on burnout have typically been demonstrated through (1) a main effects model (direct negative associations between social support and burnout) and (2) a stress-buffering model (social support buffering the negative effects of stress on burnout). While both models provide insights into functional adaptations to burnout and stress in sport, evidence for significant main and stress-buffering effects are inconsistent. Reasons for this is include: (1) testing of a singular perspective of support in empirical research, and (2) a lack of specificity when analyzing social support and burnout (e.g., adoption of global-level analyses). To address this, the purpose of the study was to test differing perspectives of social support (perceived availability of support and received support) in regards to the main and stress-buffering effects of dimensions of social support (emotional, esteem, informational, and tangible) on dimensions of burnout (reduced sense of accomplishment, devaluation, emotional and physical exhaustion). Cross-sectional data were collected from 222 athletes. Moderated hierarchical regression analyses revealed that: (1) higher levels of stress were associated with higher levels of burnout (all dimensions); (2) higher levels of perceived availability of support were associated with lower levels of reduced sense of accomplishment and devaluation (with the exception of perceived availability of emotional support upon devaluation), and (3) perceived availability of emotional support buffered the negative effects of high stress upon devaluation. There were no significant main or interactive effects for any dimensions of received support. The significant interaction suggests that higher levels of perceived availability of emotional support may result in a functional adaptation to higher stress such that individuals may be protected from higher levels of devaluation of sport.

## Introduction

Sport participation commonly involves exposure to a range of stressors (Fletcher et al., 2006; Sarkar and Fletcher, 2014). Yet the experience of stress has the potential to lead to burnout and negatively impact upon the psychological wellbeing of athletes (Udry et al., 1997; Gustafsson et al., 2017). While social support has the potential to protect athletes from deleterious adaptations to stress (e.g., from burnout: DeFreese and Smith, 2014; Lu et al., 2016), a lack of differentiated investigations have prevented researchers from developing a more nuanced understanding of how these constructs are related to one another. Such understanding would inform the design of theory-led interventions. As such, the purpose of the present study was to test differing perspectives of social support (perceived availability of support and received support) in regards to the main and stress-buffering effects of dimensions of social support (emotional, esteem, informational, and tangible) on dimensions of burnout (reduced sense of accomplishment, devaluation, emotional, and physical exhaustion).

There is an abundance of evidence demonstrating the beneficial effects of socially supportive relationships in sport (Holt and Hoar, 2006; Rees and Freeman, 2007; Lu et al., 2016). Social support has been positively associated with objective performance outcome (Freeman and Rees, 2008, 2009; Rees and Freeman, 2009, 2010), Olympic performance (Gould et al., 2002), challenge appraisals (Freeman and Rees, 2009), flow (Bakker et al., 2011), and self-confidence (Holt and Hoar, 2006; Rees and Freeman, 2007; Freeman et al., 2011), as well as lower risks for injury (Carson and Polman, 2012) and burnout (Freeman et al., 2011; DeFreese and Smith, 2013, 2014; Lu et al., 2016). Social support encompasses both structural (i.e., number and type of relationships) and functional components of interpersonal relationships (Cohen et al., 2000; Vangelisti, 2009). Functional components refer to the particular functions and purposes served by structural relationships, and there is general agreement that functional support can be categorized into dimensions of emotional support (i.e., providing a sense of comfort, security, and being loved and cared for), esteem support (i.e., bolstering ones’ esteem and sense of competence), informational support (i.e., advice and guidance), and tangible support (i.e., concrete instrumental assistance; Rees and Hardy, 2000; Freeman et al., 2011).

Functional support, and the respective dimensions of support, are often further divided into two perspectives of support: perceived availability of support (perceived support) and received support (Vangelisti, 2009; Lakey, 2010). Perceived support refers to the subjective perception of support being available from one’s friends, family, team-mates and coaches who may provide assistance, if needed (Rees and Freeman, 2010). Received support, on the other hand, refers to support actually received—the specific helping and supportive actions provided by friends, family, team-mates, and coaches (Bianco and Eklund, 2001; Rees and Freeman, 2010). Perceived and received support are considered distinct constructs (Dunkel-Schetter and Bennett, 1990), sharing as little as 12% common variance (Haber et al., 2007) and demonstrating different relationships with outcome variables (Rees and Freeman, 2007; Freeman and Rees, 2008; Uchino, 2009). Conceptualizing social support as a complex construct (perspectives: perceived, received) and multivariate (dimensions: emotional, esteem, informational, and tangible) is relevant to concerns over matching the most appropriate dimensions and perspectives of social support to the particular demands of sport-related outcomes such as burnout (Cutrona and Russell, 1990; Berg and Upchurch, 2007).

Dimensions and perspectives of social support may be particularly salient factors in protecting against stress and reducing burnout in sport (Eklund and Defreese, 2015; Gustafsson et al., 2017). In line with the psychological stress perspective (Cohen et al., 1997), individuals exposed to the demands of the sport environment might frequently encounter sport-related stressors and experience prolonged stress (Smith, 1986; Gustafsson et al., 2008). In this regard, burnout is a deleterious adaptation to stress (Raedeke et al., 2002; Ntoumanis et al., 2012). Recent reviews defined the experience of burnout as being characterized by distinct indicators, namely physical and psychological exhaustion, and a reduced sense of accomplishment and value toward sport (Eklund and Defreese, 2015; Gustafsson et al., 2017).

While stress is considered to be a key antecedent to the formation of burnout dimensions (alongside other contributing factors; Raedeke, 1997; Gustafsson et al., 2017), exposure to stressors does not necessarily lead to the experience of stress and formation of burnout, as social factors may protect against them (DeFreese and Smith, 2013). Specifically, perceived support is theorized to influence individuals’ perceived capabilities and resources to cope with stressors, thereby affecting both primary and secondary stress-appraisals (Lazarus and Folkman, 1984; Freeman and Rees, 2009). Received support is theorized to intervene in response to stress experienced (e.g., through moderating coping behaviors), which may have implications for dimensions of burnout (Cohen et al., 2000; Bianco and Eklund, 2001). Indeed, social support is typically associated with lower levels of burnout dimensions (DeFreese and Smith, 2013, 2014), and may be considered an effective resource for protecting against the deleterious effects of stress and dimensions of burnout in sport (Freeman et al., 2011; Lu et al., 2016).

There have been investigations into the relative impact of specific dimensions of social support upon global burnout (e.g., Lu et al., 2016), and there have been comparisons made between perceived and received support at a global level upon dimensions of burnout (e.g., DeFreese and Smith, 2013, 2014). However, there are limitations to using global measures. Global measures of social support and burnout ignore the possibility that certain dimensions of support might be more strongly associated with certain dimensions of burnout (DeFreese and Smith, 2013; Freeman et al., 2014; Lu et al., 2016), and there may be discrepancies in the magnitude of these contributions.

Indeed, the development of burnout is a highly individualistic experience (Gould et al., 1997; Gustafsson et al., 2007), with longitudinal evidence suggesting individual dimensions of burnout may not develop in tandem (Isoard-Gautheu et al., 2015). For example, Lundkvist et al. (2018) found that exhaustion negatively predicted devaluation across a 6-month period (after which this association faded within an 18-month sample), and argued that several models outlining the proposed development of burnout indices appear to be problematic in sport contexts. There are also theoretical grounds for expecting discrepancies in the presence and magnitude of dimensional associations between social support and burnout, as certain dimensions of support might allow for functional adaptations to certain outcomes (Cutrona and Russell, 1990). For example, certain dimensions of support might exclusively foster specific types of coping behavior in response to deleterious adaptations to stress (such as burnout; Cohen and Wills, 1985). This can only be investigated using dimensional measures of social support and, to our knowledge, Freeman et al. (2011) have been the only researchers to investigate the main effects of specific dimensions of support upon specific dimensions of burnout in sport. Freeman et al. (2011) reported that esteem support was the only significant predictor for reduced sense of accomplishment, and informational support was the only significant predictor for devaluation and for emotional and physical exhaustion. These results suggest there may indeed be discrepancies in the presence and magnitude of associations between dimensions of social support and dimensions of burnout.

Two principal models typically guide social support research: (1) the main effects model, and (2) the stress-buffering model (Cohen and Wills, 1985; Cohen et al., 2000). The main effects model proposes social support to have a direct effect on outcomes irrespective of whether individuals are under high or low levels of stress; the stress-buffering model proposes social support to be related to outcomes as a function of stress (Cohen et al., 2000; Rees and Freeman, 2007; Freeman and Rees, 2010). Although perceived support is theorized to act primarily through the main effects model and received support through the stress-buffering model (Bianco and Eklund, 2001), researchers have often found evidence to the contrary. For example, perceived support has been found to buffer the deleterious effects of stress upon outcomes (Rees and Hardy, 2004; Freeman and Rees, 2010), and researchers have cited that there is only limited evidence for received support buffering the deleterious effects of stress upon outcomes (Rees and Freeman, 2007; Rees et al., 2007; Mitchell et al., 2014). Furthermore, it seems only two studies have directly investigated the stress-buffering effects of social support in relation to burnout in sport – yet these studies only investigated dimensional stress-buffering effects of received support upon global burnout (Lu et al., 2016), and global stress-buffering of social support upon dimensions of burnout (DeFreese and Smith, 2014). In short, our understanding of the dimensional operationalization of social support upon burnout through main and stress-buffering models remains unclear (Rueger et al., 2016).

A comparison of main and stress-buffering effects for perceived versus received support warrants a further consideration with regards to method of analyses. When perceived and received support are examined separately, both tend to be associated with main and stress-buffering effects, however, when examined together different effects tend to be observed (Rees and Freeman, 2007; Freeman and Rees, 2008). It has been suggested that although perceived and received support are considered separate constructs (Wethington and Kessler, 1986; Dunkel-Schetter and Bennett, 1990; Helgeson, 1993), they may potentially influence each other and be conceptually related under certain circumstances (Uchino, 2009). Considering this, it is advisable to simultaneously examine the differential impact of perceived and received support dimensions upon outcomes, as it might provide an indication as to which perspective of support exerts greater and/or unique effects upon outcomes and under what conditions (Dunkel-Schetter and Bennett, 1990; Bianco and Eklund, 2001; Rees and Freeman, 2007; Freeman and Rees, 2010).

The purpose of the present study was to test differing perspectives of social support (perceived availability of support and received support) in regards to the main and stress-buffering effects of dimensions of social support (emotional, esteem, informational, and tangible) on dimensions of burnout (reduced sense of accomplishment, devaluation, emotional and physical exhaustion). Considering the dearth of evidence upon which to postulate fully differentiated hypotheses in line with this purpose (e.g., DeFreese and Smith, 2013; DeFreese and Smith, 2014; Lu et al., 2016), we hypothesized the following: (1) higher levels of stress would be associated with higher levels of burnout dimensions; (2) there would be differences observed between perceived and received dimensional main effects of support on dimensions of burnout; and, (3) there would be differences observed between perceived and received support dimensional stress-buffering effects on dimensions of burnout.

## Materials and Methods

### Participants

Participants were 222 athletes (122 male; mean age of 25.93 years, *SD* = 10.11 years), partaking in a range of 54 different sports (the most frequent of which were cycling, rugby, and soccer). The competitive levels of participants ranged from recreational (*n* = 58), club (*n* = 52), regional (*n* = 57), national (*n* = 36), to international (*n* = 19) standard.

### Procedure

The study was approved by a University Ethics Committee and all participants provided informed consent. An online questionnaire was constructed and disseminated opportunistically through online portals, with all questionnaire sections randomized and counter-balanced to control for order-effects.

### Measures

#### Stress

Participants were asked to indicate the degree of stress experienced by completing a 4-item measure representing four sources of sport-specific stress commonly drawn upon within the literature (e.g., Freeman and Rees, 2008, 2010): high performance concerns from others, injury concerns, stamina/fitness concerns, and doubts about current form. This approach to assessing specific stress experienced resulting from each stressor is in line with the psychological stress perspective (Cohen et al., 1997; Freeman and Rees, 2008), which focuses on whether individuals experience context-specific stress (as opposed to general stress) and not merely whether they encountered particular sport-related stressors. As developed by Freeman and Rees (2008, 2010), and given that there may be individual differences in the extent and timeliness of stress reactions (Lazarus and Folkman, 1984), the stem for each item was: “Please indicate how stressed you felt as a result of the following situations over the past two weeks.” Participants were given 2 weeks to consider their stress experienced to ensure applicability to a range of athletes and timings across different sports and to gather an estimation of levels of stress. Participants were required to respond on a 5-point Likert scale ranging from 1 (*not at all*) to 5 (*a lot*). Item responses were summed to reduce the number of models and aid clarity of interpretation by creating a total score of stress (α = 0.77).

#### Perceived Support

The 16-item Perceived Available Support in Sport Questionnaire (the PASS-Q; Freeman et al., 2011) was used to assess perceived support. The PASS-Q has demonstrated good reliability and validity indices across independent samples (Freeman et al., 2011; Boat and Taylor, 2015). The stem for the PASS-Q is: “Please indicate to what extent you have these types of support available to you.” Participants were required to respond on a 5-point Likert scale, ranging from 0 (*not at all*) to 4 (*extremely*). In line with the established factorial structure of the PASS-Q, dimensional item responses were averaged to create subscale (dimensional) scores for emotional (α *=* 0.90), esteem (α *=* 0.92), informational (α *=* 0.91), and tangible perceived support (α *=* 0.85).

#### Received Support

The 22-item Athletes’ Received Support Questionnaire (the ARSQ; Freeman et al., 2014) was used to assess received support. The ARSQ has demonstrated good reliability and validity indices across independent samples (Freeman et al., 2014). The stem for the ARSQ is: “Please indicate the frequency with which you received each type of support during the last week.” Participants were required to respond on a 5-point frequency scale, ranging from 0 (*not at all*) to 4 (*seven or more times*). In line with the established factorial structure of the ARSQ, dimensional item responses were averaged to create subscale scores for emotional (α *=* 0.89), esteem (α *=* 0.90), informational (α *=* 0.92), and tangible received support (α *=* 0.92).

#### Burnout

Dimensions of athlete burnout were assessed using the 15-item Athlete Burnout Questionnaire (ABQ; Raedeke and Smith, 2001), which has demonstrated good construct and structural validity in independent samples (Cresswell and Eklund, 2006; Raedeke and Smith, 2009; Gerber et al., 2018). The stem for the ABQ is: “Please indicate the extent to which you are currently experiencing each feeling.” Participants were required to respond on a 5-point Likert scale, ranging from 0 (*almost never*) to 4 (*almost always*). In line with the established factorial structure of the ABQ, dimensional item responses were averaged to provide subscale scores for reduced sense of accomplishment (α = 0.79), devaluation (α = 0.81), and emotional and physical exhaustion (α = 0.90).

### Analyses

The data were screened for outliers, indices of non-normality, and missing values, of which there were none. In order to compare, simultaneously, the main and stress-buffering potential for each dimension of perceived and received support upon dimensions of burnout, moderated hierarchical regression analyses were performed using a three-step process within the enter-method of regression (Cohen and Wills, 1985; Freeman and Rees, 2008). First, stress was entered at Step 1. Second, respective dimensions of perceived and received support (e.g., emotional perceived support and emotional received support) were entered at Step 2. Finally, the product terms for each support and stress (e.g., stress × emotional perceived support and stress × emotional received support) were entered at Step 3. Prior to analyses, all independent variables (stress, dimensions of perceived support, and dimensions of received support) were mean-centred (Jaccard et al., 1990). The significance of increments in explained variance in dimensions of burnout over and above that accounted for by the already-entered variables was assessed at each step.

## Results

Descriptives and bivariate correlations between all variables in the study are presented in Table 1. Stress was positively associated with all dimensions of burnout, and higher levels of perceived and received support were associated with lower levels of reduced sense of accomplishment and devaluation (except for the non-significant association between received tangible support and devaluation).

**Table 1:** Means, standard deviations, and bivariate correlations for study variables.

Variable		1	2	3	4	5	6	7	8	9	10	11	12
1	Reduced Sense of Accomplishment												
2	Devaluation	0.47**											
3	Emotional and physical exhaustion	0.23**	0.44**										
4	Stress	0.18**	0.27**	0.41**									
5	Perceived Emotional Support	–0.35**	–0.24**	0.01	–0.11								
6	Perceived Esteem Support	–0.43**	–0.23**	–0.04	–0.01	0.78**							
7	Perceived Informational Support	–0.40**	–0.25**	–0.04	–0.01	0.55**	0.77**						
8	Perceived Tangible Support	–0.38**	–0.25**	0.06	–0.06	0.70**	0.75**	0.72**					
9	Received Emotional Support	–0.22**	–0.15*	0.09	0.17*	0.54**	0.53**	0.41**	0.54**				
10	Received Esteem Support	–0.32**	–0.19**	0.05	0.04	0.49**	0.64**	0.54**	0.60**	0.84**			
11	Received Informational Support	–0.28**	–0.16*	0.09	0.14*	0.42**	0.56**	0.61**	0.59**	0.72**	0.77**		
12	Received Tangible Support	–0.26**	–0.10	0.12	0.10	0.43**	0.51**	0.52**	0.67**	0.67**	0.67**	0.84**	
	*M*	2.58	2.31	2.49	2.75	2.60	2.70	2.70	2.37	2.16	2.41	1.96	1.82
	*SD*	0.74	0.90	0.91	0.96	1.00	0.95	0.98	1.03	1.13	1.05	1.07	1.19

*N = 222; ^∗^*p* < 0.05; ^∗∗^*p* < 0.01.*

### Main and Stress-Buffering Effects for Dimensions of Perceived and Received Support Upon Dimensions of Burnout

Results from moderated hierarchical regression analyses are presented in Table 2. At Step 1, there were significant positive main effects for stress upon reduced sense of accomplishment (Cohen’s *F^2^* = 0.03, a small effect), devaluation (Cohen’s *F*2 = 0.08, a small effect), and emotional and physical exhaustion (Cohen’s *F^2^* = 0.20, a medium effect). In summary, higher levels of stress were associated with higher levels on dimensions of burnout.

**Table 2:** Moderated hierarchical regression results.

						Dependent Variable		
			RSA			DEV			EXH		
Dimension of support		Step	*ΔR^2^*	*F*	B	*ΔR^2^*	*F*	B	*ΔR^2^*	*F*	B
Emotional	1	Stress	0.03**	7.13	0.13**	0.07**	17.78	0.25**	0.17**	44.35	0.37**
	2	Perceived Support	0.12**	12.55	–0.21**	0.06**	10.85	–0.13	<0.01	14.99	0.06
		Received Support			–0.07			–0.11			–0.01
	3	Stress × PS	<0.01	7.72	<0.01	0.03*	8.12	–0.17*	<0.01	9.08	–0.05
		Stress × RS			0.05			0.01			0.01
Esteem	1	Stress	0.03**	7.13	0.13**	0.07**	17.78	0.25**	0.17**	44.35	0.37**
	2	Perceived Support	0.19**	20.76	–0.27**	0.06**	11.17	–0.15*	0.01	15.27	–0.08
		Received Support			–0.07			–0.09			0.08
	3	Stress × PS	0.01	12.95	–0.05	<0.01	6.88	–0.07	0.01	9.39	–0.08
		Stress × RS			0.08			0.01			0.03
Informational	1	Stress	0.03**	7.13	0.13**	0.07**	17.78	0.25**	0.17**	44.35	0.37**
	2	Perceived Support	0.16**	17.67	–0.25**	0.06**	11.67	–0.17*	0.01	15.23	–0.08
		Received Support			–0.08			–0.08			0.08
	3	Stress × PS	<0.01	10.81	–0.07	<0.01	7.00	–0.04	0.01	9.50	–0.10
		Stress × RS			0.05			0.03			0.08
Tangible	1	Stress	0.03**	7.13	0.13**	0.07**	17.78	0.25**	0.17**	44.35	0.37**
	2	Perceived Support	0.14**	14.63	–0.25**	0.05**	10.77	–0.24**	0.01	15.45	0.05
		Received Support			–0.04			0.04			0.04
	3	Stress × PS	0.01	9.16	–0.08	0.01	7.08	–0.13	0.01	9.80	–0.12
		Stress × RS			0.08			0.07			0.06

*N = 222; ^∗^*p* < 0.05; ^∗∗^*p* < 0.01; RSA, reduced sense of accomplishment; DEV, devaluation of sport; EXH, emotional and physical exhaustion; PS, perceived support; RS, received support. All variables standardized except for products. Products were formed from preceding (standardized) variables.*

At Step 2, there were, with the exception of a non-significant effect of perceived emotional support upon devaluation, significant negative main effects for all dimensions of perceived support upon reduced sense of accomplishment (Cohen’s *F^2^* ranging between 0.14 and 0.23, representing medium effects) and devaluation (Cohen’s *F^2^* = ranging between 0.05 and 0.06, representing small effects). For all significant effects, higher levels of support were associated with lower levels of burnout. There were no significant main effects for perceived support upon emotional and physical exhaustion, and there were no significant main effects for any dimensions of received support upon any dimensions of burnout.

**FIGURE 1:**
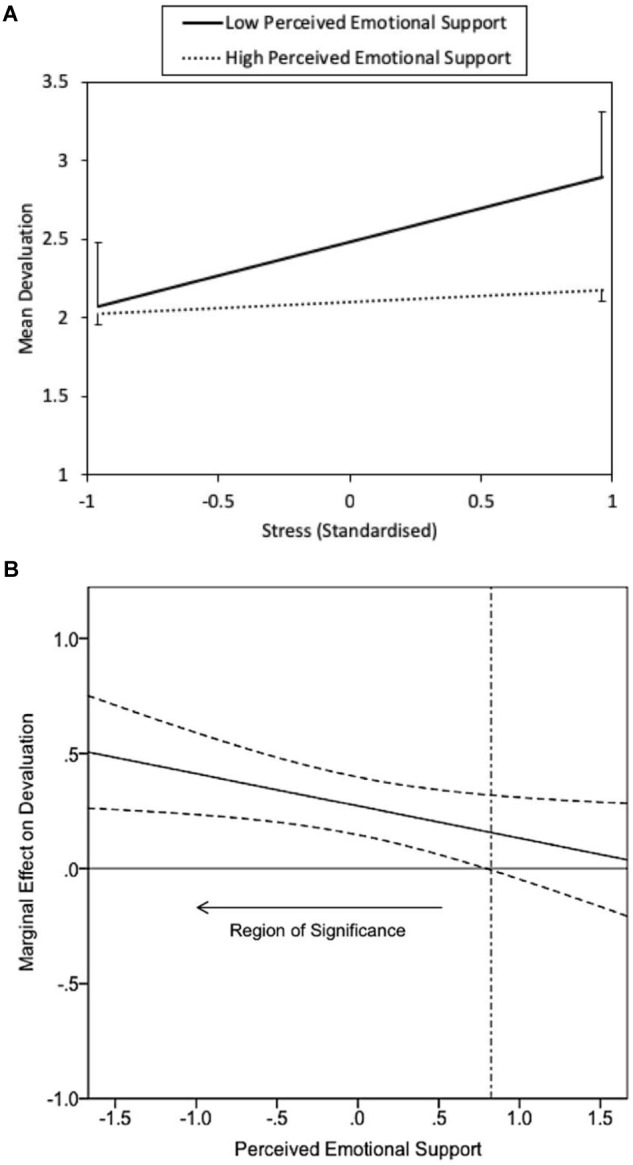
The interactive relationship between stress and perceived emotional support upon devaluation **(A)**, with simple slopes analysis **(B)**.

Finally, at Step 3 the interaction of stress x perceived emotional support explained significant additional variance in devaluation, *F* (2,217) = 8.12^∗∗^, *b* = -0.17^∗∗^, *SE* = 0.07 [-0.30, -0.04], Cohen’s *F^2^* = 0.03 (a small effect; the interaction is depicted in Figure 1A). The relationship between stress and devaluation was significantly different from zero at low (*t* = 5.10, *p* < 0.01) but not at high levels of perceived emotional support (*t* = 0.99, *p* = 0.33). Specifically, the relationship between stress and devaluation differed significantly from zero at levels of perceived emotional support less than 0.82 standard deviations above the mean (the simple slopes analysis is depicted in Figure 1B). The interaction was consistent with a stress-buffering explanation: higher levels of perceived emotional support negated the deleterious effects of higher levels of stress on devaluation (rather than burnout).

## Discussion

The purpose of the present study was to test differing perspectives of social support (perceived availability of support and received support) in regards to the main and stress-buffering effects of dimensions of social support (emotional, esteem, informational, and tangible) on dimensions of burnout (reduced sense of accomplishment, devaluation, emotional and physical exhaustion). Hypothesis 1 was supported. Stress had deleterious relationships with all dimensions of burnout, such that higher levels of stress were associated with higher levels of dimensions of burnout. These results support stress-based models of burnout (Eklund and Defreese, 2015; Gustafsson et al., 2017). Hypotheses 2 was partially supported. Differences were observed between the dimensional main effects for perceived versus received support upon dimensions of burnout. Finally, Hypothesis 3 was supported. Differences were observed between the dimensional stress-buffering effects for perceived versus received support upon dimensions of burnout.

With regards to Hypothesis 2, higher levels of perceived availability of support were associated with lower levels of reduced sense of accomplishment and devaluation (with the exception of perceived availability of emotional support upon devaluation). No effects for received support on dimensions of burnout were observed. Although we did not hypothesize directional differences to exist between these fully differentiated measures, these findings are similar to previously found associations between global (DeFreese and Smith, 2013) and dimensional social support (Freeman et al., 2011) with dimensions of burnout. The observed differences between the independent main effects for perspectives of social support when entered simultaneously upon dimensions of burnout suggest that, compared to received support, perceived support was more strongly associated with dimensions of burnout. These findings are in line with global-level social support research that demonstrates (1) higher levels of perceived support are associated with lower levels of burnout (Bianco and Eklund, 2001; Freeman et al., 2011), and (2) received support is less consistently associated with outcome variables (Rees and Hardy, 2004; Rees and Freeman, 2007; Freeman and Rees, 2008, 2009; Lakey, 2010; Boat and Taylor, 2015) such as dimensions of burnout (DeFreese and Smith, 2013, 2014).

Empirical evidence from the extant literature reports discrepancies in the magnitude of perceived support’s contributions to specific dimensions of burnout (e.g., DeFreese and Smith, 2013). Our findings support this. All perceived support dimensions had medium associations with reduced sense of accomplishment and small associations with devaluation (with the exception of perceived emotional support), however, no perceived support dimensions were associated with emotional and physical exhaustion. The medium association between perceived support and reduced sense of accomplishment suggests that knowing that different dimensions of supportive acts are available if needed might combat feelings of inefficacy and the tendency to evaluate oneself negatively in terms of performance capabilities. The small association between perceived support and devaluation suggests that knowing that different dimensions of supportive acts are available if needed might bolster ones’ concern for performance quality and encourage a more positive attitude toward sport participation (Eklund and Defreese, 2015; Gustafsson et al., 2017). In contrast, there was an absence of an association between perceived support and exhaustion. Considering that physical exhaustion is a natural part of sport, it may be worthwhile exploring if the relevance of perceived support depends on whether exhaustion is driven primarily by physical (perceived support perhaps of little relevance) or psychological (perceived support perhaps of greater relevance) causes (DeFreese and Smith, 2013).

Further to our second hypothesis, the only difference observed between dimensions of perceived support and dimensions of burnout was an absence of an association between perceived emotional support and devaluation (perceived esteem, informational, and tangible support were associated with devaluation). This dimensional difference suggests that merely increasing levels of perceived social support in a global manner may not translate directly into beneficial outcomes. Further, providing unsatisfactory forms of support may fail to result in beneficial sport-related outcomes. For example, in our study, increasing levels of perceived emotional support did not result in beneficial adaptations for devaluation. Similarly, Freeman and Rees (2009) found that the only significant dimension of perceived support associated with enhanced performance was esteem support, which was shown to have both positive associations with challenge appraisals (through perceptions of situational control), and negative associations with threat appraisals. This supports Cohen and Wills (1985) theorizing that emotional and esteem support could be most useful in a range of achievement contexts, while informational and tangible support may be more effective in particular situations (Cohen and Wills, 1985). Indeed, specific associations have been found between certain dimensions of social support and other sport-related outcomes such as self-confidence (Freeman et al., 2011), burnout (dimensionally and globally; Freeman et al., 2011; Lu et al., 2016), and performance (Rees et al., 2007).

With regards to Hypothesis 3, only perceived emotional support buffered the deleterious association of stress upon devaluation. No dimensions of received support buffered the deleterious association of stress upon burnout dimensions. The observed differences between the independent stress-buffering effects for perspectives of social support when entered simultaneously upon dimensions of burnout are in line with previous research that reports that, compared to received support, perceived support is more consistently associated with stress-buffering (Rees and Hardy, 2004; Rees et al., 2007, 2010; Freeman and Rees, 2010; Mitchell et al., 2014). Perceived support, compared to received support, may also be more consistently related to beneficial outcomes. Perhaps the consistent perceived availability of support over time may lead to the formation of “trait-like” support profiles. In turn, this may facilitate persistent perceptions of support resource availability and control in individuals during times of stress (compared to received support which may be more context-dependent; Freeman and Rees, 2009; Uchino, 2009). Conversely, as seen in instances where social support fails to be beneficial (or even harmful; Schwarzer and Leppin, 1991; Reynolds and Perrin, 2004; Brock and Lawrence, 2009; Kellezi and Reicher, 2012), received support may unintentionally undermine recipients’ perceptions of competency or autonomy, potentially triggering experiences of stress and/or feelings of embarrassment (e.g., Bolger and Amarel, 2007; Hassell et al., 2010). Consistent with a resiliency perspective (Sarkar and Fletcher, 2014), perceived support may thus allow for more functional adaptations to stress and dimensions of burnout, as knowing that support is available if needed may increase ones’ perceived social resources and abilities to cope (Lazarus and Folkman, 1984; Bianco and Eklund, 2001), thereby resulting in more challenge and less threat-based stress appraisals (Freeman and Rees, 2009). As such, perceived support may result in more functional adaptations to dimensions of burnout by reducing the experience of stress (Gustafsson et al., 2017), as well as improving one’s concern for performance quality and/or encouraging a more positive attitude toward sport participation (i.e., reducing devaluation; Raedeke et al., 2002).

Further to our third hypothesis, the only difference observed between dimensions of perceived support and dimensions of burnout was perceived emotional support buffering the deleterious association of stress on devaluation (perceived esteem, informational, and tangible support did not exhibit any stress buffering effects on any dimensions of burnout). This is only somewhat in line with previous findings, as Freeman and Rees (2010) found stress-buffering effects for perceived emotional, esteem and informational support dimensions upon self-confidence. Furthermore, our study found that no dimensions of received support buffered the deleterious associations of stress upon burnout dimensions. Although this is in line with evidence showing that received support dimensions may fail to exhibit stress-buffering effects upon global burnout (Lu et al., 2016), this contrasts with evidence showing global received support to exhibit stress-buffering effects upon self-confidence and performance (Rees and Freeman, 2007; Freeman and Rees, 2008). While the dimensional stress-buffering effect observed in this study provides empirical evidence for a stress-buffering effect in sport more generally, it highlights the importance of adopting multivariate conceptualizations of social support and outcomes, such as burnout (Lakey and Cronin, 2008; Rueger et al., 2016; Lundkvist et al., 2018). Indeed, it could be that knowing emotional support is available if needed, particularly during times of high levels of stress, may lead to the ideal sort of emotion-focused coping (e.g., Cutrona and Russell, 1990) needed when an athlete feels a detached and cynical attitude toward their performance quality and/or sport (Rees and Hardy, 2004). It may, therefore, be that merely increasing levels of social support irrespective of an athlete’s social support or burnout-related needs may not translate directly into functional adaptations to stress (i.e., stress-buffering), and there may even be risks associated with providing unsatisfactory forms of support (i.e., resulting in deleterious adaptations to stress; Freeman and Rees, 2008).

The present study has several strengths. First, questionnaires developed in social and health psychology (e.g., SSQ; Sarason et al., 1987) have often been used in sport, and their utility in sport has been questioned as they do not necessarily reflect the specific forms of support that athletes require (Rees et al., 1999; Holt and Hoar, 2006). Therefore, our use of dimensional social support and burnout measures derived for the sport context (e.g., Raedeke and Smith, 2001; Freeman et al., 2011, 2014) reduces concerns over measurement error (Dunkel-Schetter and Bennett, 1990; Gerber et al., 2018), together with providing more sensitive tests for moderation (Uchino et al., 2012; Rueger et al., 2016). Second, to the best of our knowledge, our study is the first to investigate both main and stress buffering effects while using recommended multivariate conceptualizations of both social support and burnout (e.g., Freeman et al., 2011, 2014; Lundkvist et al., 2018). Fully differentiated investigations allow researchersk to determine the relative impact of different perspectives of support (i.e., received versus perceived) and specific supportivek acts (i.e., dimensions) upon adaptations to stress and other sport-related outcomes (Freeman and Rees, 2010; Hassell et al., 2010). Developing a more nuanced understanding of *how* different perspectives and dimensions of social support (and other contributing factors) influence functional adaptations to stress and other sport-related outcomes highlights an important area for future research. Such investigations will advance our understanding of stress and athlete psychological functioning more generally, and inform the design of interventions focussed on specific perspectives and dimensions of supportive acts (Freeman and Rees, 2009; Thoits, 2011).

Some limitations of the present study should also be noted. First, the use of a cross-sectional design prevents any causal inferences from being made. Second, while dimensional investigations into perceived versus received support and burnout allows for evaluations of the effects of specific support perspectives and specific supportive acts (i.e., dimensions; Cutrona and Russell, 1990; Raedeke and Smith, 2009), it does have several disadvantages: (1) it reduces parsimony for determining the differences between perspectives and dimensions of support (Rees and Freeman, 2007), and; (2) running multiple stress-buffering models may increase the risk of Type 1 Error (although this number of models is similar to those computed in previous social support research; DeFreese and Smith, 2014). Relatedly, an examination of gendered effects were beyond the scope of the current study. There is some evidence that a non-significant (Lai and Wiggins, 2003) to small gendered effect may exist for both work (Purvanova and Muros, 2010) and sport related burnout (Cremades and Wiggins, 2008; Isoard-Gautheu et al., 2015), and this may be an interesting avenue for future research to explore. Furthermore, due to the range of sports and athletes recruited, participants may have been at different stages of their competitive seasons and/or been injured, and it is therefore possible that our interpretation of the analysis could have been influenced had such demographic data been collected (Cresswell and Eklund, 2005; Quested and Duda, 2011). Future research may consider incorporating such variables in analyses.

Considering the above, more research is needed into the underlying mechanisms of why and under what conditions certain perspectives and/or dimensions of social support are more strongly associated with stress and particular dimensions of burnout. For example, Lu et al. (2016) found that under conditions of low stress, athletes with higher (lower) levels of resilience but low (high) levels of informational support were less prone to global burnout than those who were low in both resilience and informational social support. This suggests that single moderators may fail to fully capture the complexity of social support’s stress-buffering effects (Smith et al., 1990). It is reasonable, therefore, to think that specific perspectives and/or dimensions of social support may interact in a conjunctive manner (Smith et al., 1990) with other socio-contextual moderators to influence the stress-burnout relationship. To provide an example, Social Identity Theory (Tajfel and Turner, 1979; Turner et al., 1987) posits the experience of sport-related stress and social support to be bound-up within the social dynamics of group life (Rees et al., 2015), both in terms of accentuating or alleviating the effects of stress (Lazarus and Folkman, 1984) and social support (Turner, 1991; Rees et al., 2013).

## Conclusion

In conclusion, the findings from the present study highlight the unique differences observed between differing perspectives of social support (perceived availability of support and received support) in regards to the main and stress-buffering effects of dimensions of social support (emotional, esteem, informational, and tangible) on dimensions of burnout (reduced sense of accomplishment, devaluation, emotional and physical exhaustion). Our findings help address an important gap in the literature by showing that higher levels of perceived availability of emotional support may result in a functional adaptation to higher stress such that individuals may be protected from higher levels of devaluation of sport.

## Data Availability

The raw data supporting the conclusions of this manuscript will also be made available, without undue reservation, to any qualified researcher.

## Ethics Statement

This study was carried out in accordance with the recommendations of the University of Stirling’s Research Proposal Ethics Checklist (General University Ethics Panel), with written informed consent from all subjects. All subjects gave written informed consent in accordance with the Declaration of Helsinki. The protocol was approved by the University of Stirling’s General University Ethics Panel.

## Author Contributions

Both authors contributed equally to the design, data collection, analysis and writing of this manuscript.

## Conflict of Interest Statement

The authors declare that the research was conducted in the absence of any commercial or financial relationships that could be construed as a potential conflict of interest.

## References

[B1] Bakker A. B. Oerlemans W. Demerouti E. Slot B. B. Ali D. M. (2011). Flow and performance: a study among talented Dutch soccer players. *Psychol. Sport Exerc.* 12 442–450. 10.1016/j.psychsport.2011.02.003

[B2] Berg C. A. Upchurch R. (2007). A developmental-contextual model of couples coping with chronic illness across the lifespan. *Psychol. Bull.* 133 920–954. 10.1037/0033-2909.133.6.920 17967089

[B3] Bianco T. Eklund R. C. (2001). Conceptual considerations for social support research in sport and exercise settings: the case of sport injury. *J. Sport Exerc. Psychol.* 23 85–107. 10.1123/jsep.23.2.85

[B4] Boat R. Taylor I. M. (2015). Patterns of change in psychological variables leading up to competition in superior versus inferior performers. *J. Sport Exerc. Psychol.* 37 244–256. 10.1123/jsep.2014-0216 26265338

[B5] Bolger N. Amarel D. (2007). Effects of social support visibility on adjustment to stress: experimental evidence. *J. Pers. Soc. Psychol.* 92 458–475. 10.1037/0022-3514.92.3.458 17352603

[B6] Brock R. L. Lawrence E. (2009). Too much of a good thing: underprovision versus overprovision of partner support. *J. Family Psychol.* 23 181–192. 10.1037/a0015402 19364212 PMC2776033

[B7] Carson F. Polman R. (2012). Experiences of professional rugby union players returning to competition following anterior cruciate ligament reconstruction. *Phys. Ther. Sport* 13 35–40. 10.1016/j.ptsp.2010.10.007 22261429

[B8] Cohen S. Gottlieb B. H. Underwood L. G. (2000). “Social relationships and health,” in *Social Support Measurement and Intervention: A Guide for Health and Social Scientists* eds Cohen S. Underwood L. G. Gottlieb B. H. (New York, NY: Oxford University Press) 3–25.

[B9] Cohen S. Kessler R. C. Underwood-Gordon L. G. (1997). “Strategies for measuring stress in studies of psychiatric and physical disorders,” in *Measuring Stress: A Guide for Health and Social Scientists* eds Cohen S. Kessler R. C. Underwood-Gordon L. G. (Oxford: Oxford University Press) 3–26.

[B10] Cohen S. Wills T. A. (1985). Stress, social support and the buffering hypothesis. *Psychol. Bull.* 98 310–357. 10.1037//0033-2909.98.2.310 3901065

[B11] Cremades J. Wiggins M. (2008). *Direction and Intensity of Trait Anxiety as Predictors of Burnout Among Collegiate Athletes*. Available at: http://www.athleticinsight.com/Vol10Iss2/TraitAnxiety.htm (accessed June 12 2019).

[B12] Cresswell S. L. Eklund R. C. (2005). Changes in athlete burnout and motivation over a 12-week league tournament. *Med. Sci. Sports Exerc.* 37 1957–1966. 10.1249/01.mss.0000176304.14675.32 16286867

[B13] Cresswell S. L. Eklund R. C. (2006). The convergent and discriminant validity of burnout measures in sport: a multi-trait/multi-method analysis. *J. Sports Sci.* 24 209–220. 10.1080/02640410500131431 16368631

[B14] Cutrona C. E. Russell D. W. (1990). “Type of social support and specific stress: toward a theory of optimal matching,” in *Social Support: An Interactional View* eds Sarason SUFFIXI B. R. Sarason G. Pierce G. R. (New York, NY: Wiley) 319–336.

[B15] DeFreese J. D. Smith A. L. (2013). Teammate social support, burnout, and self-determined motivation in collegiate athletes. *Psychol. Sport Exerc.* 14 258–265. 10.1016/j.psychsport.2012.10.009

[B16] DeFreese J. D. Smith A. L. (2014). Athlete social support, negative social interactions and psychological health across a competitive sport season. *J. Sport Exerc. Psychol.* 36 619–630. 10.1123/jsep.2014-0040 25602144

[B17] Dunkel-Schetter C. Bennett T. L. (1990). “Differentiating the cognitive and behavioral aspects of social support,” in *Social Support: An Interactional View* eds Sarason SUFFIXI B. R. Sarason G. Pierce G. R. (New York, NY: Wiley) 267–296.

[B18] Eklund R. C. Defreese J. D. (2015). Athlete burnout: what we know, what we could know, and how we can find out more. *Int. Appl. Sci.* 27 63–75. 10.24985/ijass.2015.27.2.63

[B19] Fletcher D. Hanton S. Mellalieu S. (2006). “An organizational stress review: Conceptual and theoretical issues in competitive sport,” in *Literature Reviews in Sport Psychology* eds Hanton S. Mellalieu S. D. (Hauppage, NY: Nova Science) 321–375.

[B20] Freeman P. Coffee P. Moll T. Rees T. Sammy N. (2014). The ARSQ: the athletes’ received support questionnaire. *J. Sport Exerc. Psychol.* 36 189–202. 10.1123/jsep.2013-0080 24686955

[B21] Freeman P. Coffee P. Rees T. (2011). The PASS-Q: the perceived available support in sport questionnaire. *J. Sport Exerc. Psychol.* 33 54–74. 10.1123/jsep.33.1.54 21451171

[B22] Freeman P. Rees T. (2008). The effects of perceived and received support on objective performance outcome. *Eur. J. Sport Sci.* 8 359–368. 10.1080/17461390802261439

[B23] Freeman P. Rees T. (2009). How does perceived support lead to better performance? An examination of potential mechanisms. *J. Appl. Sport Psychol.* 21 429–441. 10.1080/10413200903222913

[B24] Freeman P. Rees T. (2010). Perceived social support from team-mates: direct and stress-buffering effects on self-confidence. *Eur. J. Sport Sci.* 10 59–67. 10.1080/17461390903049998

[B25] Gerber M. Gustafsson H. Seelig H. Kellmann M. Ludyga S. Colledge F. (2018). Usefulness of the Athlete Burnout Questionnaire (ABQ) as a screening tool for the detection of clinically relevant burnout symptoms among young elite athletes. *Psychol. Sport Exerc.* 39 104–113. 10.1016/j.psychsport.2018.08.005

[B26] Gould D. Greenleaf C. Chung Y. Guinan D. (2002). A survey of U.S. Atlanta and Nagano Olympians: variables perceived to influence performance. *Res. Q. Exerc. Sport* 73 175–186. 10.1080/02701367.2002.10609006 12092892

[B27] Gould D. Udry E. Tuffey S. Loehr J. (1997). Burnout in competitive junior tennis players: III. individual differences in the burnout experience. *Sport Psychol.* 11 257–276. 10.1123/tsp.11.3.257

[B28] Gustafsson H. DeFreese J. D. Madigan D. J. (2017). Athlete burnout: review and recommendations. *Curr. Opin. Psychol.* 16 109–113. 10.1016/j.copsyc.2017.05.002 28813331

[B29] Gustafsson H. Hassmen P. Kenta G. Johansson M. (2008). A qualitative analysis of burnout in elite Swedish athletes. *Psychol. Sport Exerc.* 9 800–816. 10.1016/j.psychsport.2007.11.004

[B30] Gustafsson H. Kenttä G. Hassmén P. Lundqvist C. Durand-Bush N. (2007). The process of burnout: a multiple case study of three elite endurance athletes. *Int. J. Sport Psychol.* 38 388–416.

[B31] Haber M. Cohen J. Lucas T. Baltes B. (2007). The relationship between self?-reported received and perceived social support. *Am. J. Commun. Psychol.* 39 133–144. 10.1007/s10464-007-9100-9 17308966

[B32] Hassell K. Sabiston C. M. Bloom G. A. (2010). Exploring the multiple dimensions of social support among elite female adolescent swimmers. *Int. J. Sport Psychol.* 41 340–359.

[B33] Helgeson V. S. (1993). Two important distinctions in social support: kind of support and perceived versus received. *J. Appl. Soc. Psychol.* 23 825–845. 10.1111/j.1559-1816.1993.tb01008.x

[B34] Holt N. L. Hoar S. D. (2006). “The multidimensional construct of social support,” in *Literature Reviews in Sport Psychology* eds Hanton S. Mellalieu S. D. (New York, NY: Nova Science) 1–27.

[B35] Isoard-Gautheu S. Guillet-Descas E. Gaudreau P. Chanal J. (2015). Development of burnout perceptions during adolescence among high-level athletes: a developmental and gendered perspective. *J. Sport Exerc. Psychol.* 37 436–448. 10.1123/jsep.2014-0251 26442773

[B36] Jaccard J. Turrisi R. Wan C. K. (1990). *Interaction Effects in Multiple Regression.* Newbury Park, CA: Sage. 10.1207/s15327906mbr2504_4 26820822

[B37] Kellezi B. Reicher S. D. (2012). “Social cure or social curse? The psychological impact of extreme events during the Kosovo conflict,” in *The Social Cure* eds Jetten J. Haslam C. Haslam S. A. (London: Psychology Press).

[B38] Lai C. Wiggins M. (2003). Burnout perceptions over time in NCAA Division I soccer players. *Int. Sports J.* 7 120–127.

[B39] Lakey B. (2010). “Basic research in social support suggests new strategies for intervention,” in *Social Psychological Foundations of Clinical Psychology* eds Maddux J. E. Tangey P. (New York, NY: Guildford Publications) 177–194.

[B40] Lakey B. Cronin A. (2008). “Low social support and major depression: Research, theory and methodological issues,” in *Risk Factors for Depression* eds Dobson K. S. Dozois D. (San Diego: Academic Press) 385–408. 10.1016/b978-0-08-045078-0.00017-4

[B41] Lazarus R. S. Folkman S. (1984). *Stress Appraisal and Coping.* New York, NY: Springer.

[B42] Lu F. J. H. Lee W. P. Chang Y. Chou C. Hsu Y. Lin J. (2016). Interaction of athletes’ resilience and coaches’ social support on the stress-burnout relationship: a conjunctive moderation perspective. *Psychol. Sport Exerc.* 22 202–209. 10.1016/j.psychsport.2015.08.005

[B43] Lundkvist E. Gustafsson H. Davis P. A. Holmström S. Lemyre N. Ivarsson A. (2018). The temporal relations across burnout dimensions in athletes. *Scand. J. Med. Sci. Sports* 28 1215–1226. 10.1111/sms.13000 29087026

[B44] Mitchell I. Evans L. Rees T. Hardy L. (2014). Stressors, social support and the buffering hypothesis: effects on psychological responses of injured athletes. *Br. J. Health Psychol.* 19 486–508. 10.1111/bjhp.12046 23621677

[B45] Ntoumanis N. Taylor I. M. Thøgersen-Ntoumani C. (2012). A longitudinal examination of coach and peer motivational climates in youth sport: implications for moral attitudes, well-being, and behavioral investment. *Dev. Psychol.* 48 213–223. 10.1037/a0024934 21787071

[B46] Purvanova R. K. Muros J. P. (2010). Gender differences in burnout: a meta-analysis. *J. Vocat. Behav.* 77 168–185. 10.1016/j.jvb.2010.04.006

[B47] Quested E. Duda J. L. (2011). Antecedents of burnout among elite dancers: a longitudinal test of basic needs theory. *Psychol. Sport Exerc.* 12 159–167. 10.1016/j.psychsport.2010.09.003

[B48] Raedeke T. D. (1997). Is athlete burnout more than stress? A commitment perspective. *J. Sport Exerc. Psychol.* 19 396–417. 10.1123/jsep.19.4.396

[B49] Raedeke T. D. Lunney K. Venables K. (2002). Understanding athlete burnout: coach perspectives. *J. Sport Behav.* 25 181–206. 10.1177/0033294117698465 28558533

[B50] Raedeke T. D. Smith A. L. (2001). Development and preliminary validation of an athlete burnout measure. *J. Sport Exerc. Psychol.* 23 281–306. 10.1123/jsep.23.4.281 28682196

[B51] Raedeke T. D. Smith A. L. (2009). *The Athlete Burnout Questionnaire Manual.* Morgantown, WV: West Virginia University.

[B52] Rees T. Freeman P. (2007). The effects of perceived and received support on self-confidence. *J. Sports Sci.* 25 1057–1065. 10.1080/02640410600982279 17497407

[B53] Rees T. Freeman P. (2009). Social support moderates the relationship between stressors and task performance through self-efficacy. *J. Soc. Clin. Psychol.* 28 244–263. 10.1521/jscp.2009.28.2.244

[B54] Rees T. Freeman P. (2010). Social support and performance in a golf-putting experiment. *Sport Psychol.* 24 333–348. 10.1123/tsp.24.3.333

[B55] Rees T. Hardy L. (2000). An investigation of the social support experiences of high-level sports performers. *Sport Psychol.* 14 327–347. 10.1123/tsp.14.4.327

[B56] Rees T. Hardy L. (2004). Matching social support with stressors: effects on factors underlying performance in tennis. *Psychol. Sport Exerc.* 5 319–337. 10.1016/S1469-0292(03)00018-9

[B57] Rees T. Hardy L. Freeman P. (2007). Stressors, social support, and effects upon performance in golf. *J. Sports Sci.* 25 33–42. 10.1080/02640410600702974 17127579

[B58] Rees T. Haslam S. A. Coffee P. Lavallee D. (2015). A social identity approach to sport psychology: principles, practise and prospects. *Sports Med.* 45 1083–1096. 10.1007/s40279-015-0345-4 26092187

[B59] Rees T. Ingledew D. K. Hardy L. (1999). Social support dimensions and components of performance in tennis. *J. Sports Sci.* 17 421–429. 10.1080/026404199365948 10413270

[B60] Rees T. Mitchell I. Evans L. Hardy L. (2010). Stressors, social support and psychological responses to sport injury in high and low-performance standard participants. *Psychol. Sport Exerc.* 11 505–512. 10.1016/j.psychsport.2010.07.002

[B61] Rees T. Salvatore J. Coffee P. Haslam S. A. Sargent A. Dobson T. (2013). Reversing downward performance spirals. *J. Exp. Soc. Psychol.* 49 400–403. 10.1016/j.jesp.2012.12.013

[B62] Reynolds J. S. Perrin N. A. (2004). Mismatches in social support and psychosocial adjustment to breast cancer. *Health Psychol.* 23 425–430. 10.1037/0278-6133.23.4.425 15264980

[B63] Rueger S. Y. Malecki C. K. Pyun Y. Aycock C. Coyle S. (2016). A meta-analytical review of the association between perceived social support and depression in childhood and adolescence. *Psychol. Bull.* 142 1017–1067. 10.1037/bul0000058 27504934

[B64] Sarason I. G. Sarason B. R. Shearin E. N. Pierce G. R. (1987). A brief measure of social support: practical and theoretical implications. *J. Soc. Pers. Relat.* 4 497–510. 10.1177/0265407587044007 22551428

[B65] Sarkar M. Fletcher D. (2014). Psychological resilience in sport performers: a review of stressors and protective factors. *J. Sports Sci.* 32 1419–1434. 10.1080/02640414.2014.901551 24716648

[B66] Schwarzer R. Leppin A. (1991). Social support and health: a theoretical and empirical overview. *J. Soc. Pers. Relat.* 8 99–127. 10.1177/0265407591081005

[B67] Smith R. E. (1986). Toward a cognitive-affective model of athletic burnout. *J. Sport Psychol.* 8 36–50. 10.1123/jsp.8.1.36

[B68] Smith R. E. Smoll F. L. Ptacek J. T. (1990). Conjunctive moderator variables in vulnerability and resiliency research: life stress, social support, and coping skills, and adolescent sport injuries. *J. Pers. Soc. Psychol.* 58 360–370. 10.1037//0022-3514.58.2.360 2319448

[B69] Tajfel H. Turner J. C. (1979). “An integrative theory of intergroup conflict,” in *The Social Psychology of Intergroup Relations* eds Austin W. G. Worchel S. (Monterey, CA: Brooks-Cole) 33–47.

[B70] Thoits P. A. (2011). Mechanisms linking social ties and support to physical and mental health. *J. Health Soc. Behav.* 52 145–161. 10.1177/0022146510395592 21673143

[B71] Turner J. C. (1991). *Social Influence.* Pacific Grove, CA: Brooks-Cole.

[B72] Turner J. C. Hogg M. A. Oakes P. J. Reicher S. D. Wetherell M. S. (1987). *Rediscovering the Social Group: A Self-Categorization Theory.* Oxford: Blackwell.

[B73] Uchino B. N. (2009). Understanding the links between social support and physical health: a lifespan perspective with emphasis on the separability of perceived and received support. *Perspect. Psychol. Sci.* 4 236–255. 10.1111/j.1745-6924.2009.01122.x 26158961

[B74] Uchino B. N. Bowen K. Carlisle M. Birmingham W. (2012). What are the psychological pathways linking social support to health outcomes? A visit with the ”ghosts” of research past, present, and future. *Soc. Sci. Med.* 74 949–957. 10.1016/j.socscimed.2011.11.023 22326104 PMC3298603

[B75] Udry E. Gould D. Bridges D. Tuffey S. (1997). People helping people? Examining the social ties of athletes coping with burnout and injury stress. *J. Sport Exerc. Psychol.* 19 368–395. 10.1123/jsep.19.4.368

[B76] Vangelisti A. L. (2009). Challenges in conceptualizing social support. *J. Soc. Pers. Relat.* 26 39–51. 10.1177/0265407509105520

[B77] Wethington E. Kessler R. C. (1986). Perceived support, received support, and adjustment to stressful life events. *J. Health Soc. Behav.* 27 78–89. 10.2307/2136504 3711634

